# Unraveling the New Perspectives on Antimicrobial Hydrogels: State-of-the-Art and Translational Applications

**DOI:** 10.3390/gels9080617

**Published:** 2023-07-29

**Authors:** Miguel A. Ortega, Diego De Leon-Oliva, Diego Liviu Boaru, Oscar Fraile-Martinez, Cielo García-Montero, Raul Diaz, Santiago Coca, Silvestra Barrena-Blázquez, Julia Bujan, Natalio García-Honduvilla, Miguel A. Saez, Melchor Álvarez-Mon, Jose V. Saz

**Affiliations:** 1 Department of Medicine and Medical Specialities, Faculty of Medicine and Health Sciences, University of Alcalá, 28801 Alcala de Henares, Spain; miguel.angel.ortega92@gmail.com (M.A.O.); diego.leon@edu.uah.es (D.D.L.-O.); diego.boaru@edu.uah.es (D.L.B.); oscarfra.7@hotmail.com (O.F.-M.); cielo.gmontero@gmail.com (C.G.-M.); s.coca@uah.es (S.C.); mjulia.bujan@uah.es (J.B.); natalio.garcia@uah.es (N.G.-H.); msaega1@oc.mde.es (M.A.S.); mademons@gmail.com (M.Á.-M.); 2 Ramón y Cajal Institute of Sanitary Research (IRYCIS), 28034 Madrid, Spain; raul.diazp@uah.es (R.D.); josev.saz@uah.es (J.V.S.); 3 Department of Surgery, Medical and Social Sciences, Faculty of Medicine and Health Sciences, University of Alcalá, 28801 Alcala de Henares, Spain; 4 Department of Nursing and Physiotherapy, Faculty of Medicine and Health Sciences, University of Alcalá, 28801 Alcalá de Henares, Spain; 5 Pathological Anatomy Service, Central University Hospital of Defence-UAH Madrid, 28801 Alcala de Henares, Spain; 6 Immune System Diseases-Rheumatology Service, Central University Hospital of Defence-UAH Madrid, 28801 Alcala de Henares, Spain; 7 Department of Biomedicine and Biotechnology, Faculty of Medicine and Health Sciences, University of Alcalá, 28801 Alcala de Henares, Spain

**Keywords:** antimicrobial gels, antibiotics, drug delivery, wound healing, tissue regeneration

## Abstract

The growing impact of infections and the rapid emergence of antibiotic resistance represent a public health concern worldwide. The exponential development in the field of biomaterials and its multiple applications can offer a solution to the problems that derive from these situations. In this sense, antimicrobial hydrogels represent a promising opportunity with multiple translational expectations in the medical management of infectious diseases due to their unique physicochemical and biological properties as well as for drug delivery in specific areas. Hydrogels are three-dimensional cross-linked networks of hydrophilic polymers that can absorb and retain large amounts of water or biological fluids. Moreover, antimicrobial hydrogels (AMH) present good biocompatibility, low toxicity, availability, viscoelasticity, biodegradability, and antimicrobial properties. In the present review, we collect and discuss the most promising strategies in the development of AMH, which are divided into hydrogels with inherent antimicrobial activity and antimicrobial agent-loaded hydrogels based on their composition. Then, we present an overview of the main translational applications: wound healing, tissue engineering and regeneration, drug delivery systems, contact lenses, 3D printing, biosensing, and water purification.

## 1. Introduction to Antimicrobial Hydrogels

Infections and antimicrobial drug resistance are significant global concerns that have far-reaching implications for public health [1]. The rise in drug-resistant bacteria has led to reduced treatment options and increased morbidity and mortality rates, imposing a substantial economic burden on healthcare systems [2,3,4]. Furthermore, the challenges posed by drug-resistant infections undermine medical advancements and routine procedures, such as surgeries and organ transplants [5,6]. The global spread of drug-resistant bacteria calls for collaborative efforts to contain their transmission and develop effective solutions. Addressing the global concern of infections and bacterial drug resistance requires prudent antibiotic use and robust infection prevention [7]. Additionally, investing in research and development is vital for discovering new antimicrobial agents and alternative treatment strategies such as antimicrobial gels. 

The term “hydrogel” refers to a three-dimensional (3D) porous cross-linked network with solid-like properties that can retain an extensive amount of water or biological fluids while maintaining structural and functional integrity under a variety of environmental conditions or when subjected to external stresses, which can include mechanical forces, light, temperature, and electric or magnetic fields [8,9]. Hydrogels are composed of natural polymers, synthetic polymers, or a combination of both and, in general terms, present good biocompatibility due to their biochemical similarities to the human extracellular matrix (ECM), e.g.,: high water content [10,11]. Other advantageous properties are low toxicity, availability, viscoelasticity, and biodegradability, which allow for comfortable application and adherence to various anatomical surfaces, facilitating targeted treatment [12]. In particular, antimicrobial hydrogels (AMHs) can have inherent antimicrobial activity or be loaded with metal and metal oxide nanoparticles, antibiotics, antimicrobial peptides (AMPs), or biological extracts [13,14]. With their gel-like structure, AMHs provide a 3D framework that facilitates sustained drug release, enabling localized and controlled delivery of antimicrobial substances. 

**Figure 1:**
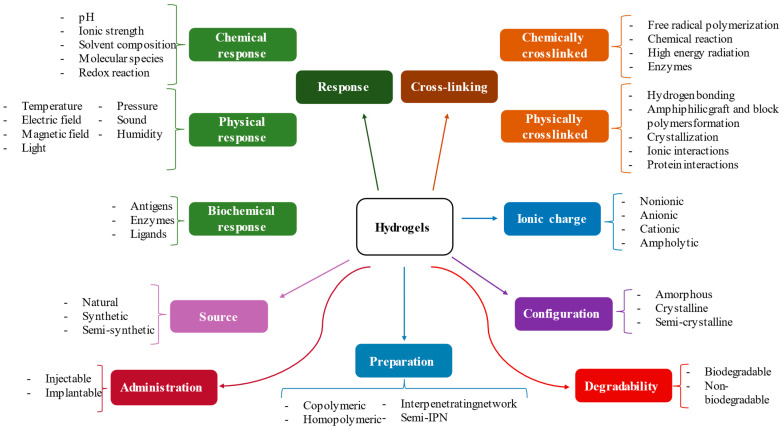
Classification of hydrogels by different criteria.

Hydrogels can be classified according to different criteria, i.e., source, cross-linking mechanism, ionic charge, preparation, or stimuli response (see Figure 1) [14,15]. The classification of AMHs according to the source of origin is determined by the materials used to create the hydrogels: Natural hydrogels derive from naturally occurring materials such as proteins, polysaccharides, and their combinations; synthetic hydrogels are created by chemical synthesis using various monomers and crosslinking agents; and hybrid hydrogels are a combination of both in order to improve existing formulations. The classification of hydrogels will be discussed in depth in Section 2.

Hydrogel synthesis typically involves the hydrolysis and condensation of selected precursors, leading to the formation of a solid nanostructured network [16]. Cross-linking agents or physical techniques are employed to connect polymer chains, resulting in the formation of a 3D structure [17]. On the one hand, chemical cross-linking involves the use of specific agents that establish covalent bonds between polymer chains, leading to permanent and strong interactions [18]. On the other hand, physical cross-linking relies on interactions such as molecular entanglements, ionic bonds, hydrophobic forces, and hydrogen bonds, which are weak, reversible, and influenced by factors such as temperature, pH, or ion-sensitive interactions [19]. 

Due to their unique properties, AMHs are used in wide-ranging biomedical applications [14,20,21]. They are utilized as wound dressings to prevent infections and promote healing [22], as scaffolds in tissue engineering to prevent microbial contamination during regeneration [23], and as drug delivery systems for controlled release of antimicrobial agents [24]. Additionally, they are used as coatings on medical implants to reduce the risk of infections. Moreover, in the field of 3D printing, antimicrobial hydrogels can be used to fabricate customized medical implants, wound dressings, and tissue scaffolds with inherent antimicrobial properties [25]. In biosensing, antimicrobial hydrogels can serve as sensing elements [26,27] and have also been explored for water purification applications [28]. AMHs provide targeted antimicrobial treatment, minimize systemic side effects, and contribute to improved patient outcomes in various biomedical settings as well as environmental sustainability. Finally, AMHs represent a promising therapeutic approach to combating bacterial resistance, as they deliver antimicrobial agents directly to the site of infection and maintain higher concentrations of the drug at the target site, potentially increasing efficacy while minimizing exposure to the rest of the organism [29].

Therefore, in this review, we aim to collect and summarize the state-of-the-art knowledge on antimicrobial gels and explore their potential translational applications in the field.

## 2. Classification of Antimicrobial Hydrogels

As mentioned in the introduction, AMHs can be classified according to different criteria (see Figure 1). Due to the objective of this review, we will explain the classification of the most commonly used AMHs based on their composition, which allows researchers and practitioners to select the most suitable gel type for specific applications, considering factors such as antimicrobial spectrum, biocompatibility, stability, and desired release kinetics.

### 2.1. Hydrogels with Inherent Antimicrobial Activity

Hydrogels with intrinsic antibacterial activity pertain to polymers within these hydrogels that possess inherent antimicrobial properties. These hydrogels, which have emerged as innovative antimicrobial agents, overcome conventional limitations. However, the comprehensive understanding of the bactericidal properties of these hydrogels is still not fully elucidated. The primary categories of these hydrogels include the following.

#### 2.1.1. Peptide-Based Hydrogels

Antimicrobial peptides (AMPs) are innate defense molecules found in multicellular organisms offering protection against competing pathogenic microbes [30,31]. Their broad-spectrum antimicrobial activity, including Gram-positive and Gram-negative bacteria, fungi, and viruses, is attributed to their ability to disrupt microbial cell membranes or interfere with intracellular processes. With their amphipathic properties, AMPs selectively interact with microbial membranes while preserving host cells. In addition to antimicrobial effects, AMPs contribute to immunomodulation and wound healing [32,33]. AMPs are currently being investigated as promising alternatives to traditional antibiotics, specifically for combating drug-resistant bacteria.

Certain antimicrobial peptides have the capability to spontaneously self-assemble into supramolecular hydrogels, which often result in an improved antimicrobial efficacy [34]. In this sense, Salick et al. developed a hydrogel based on a 20 amino acid lysine-rich amphiphilic peptide, which acquires amphiphilic β-hairpin conformation in medium with salts and rapidly self-assembles, leading to hydrogel formation [35]. MAX1 hydrogels exhibit activity against both Gram-negative bacteria, such as *E. coli* and *K. pneumoniae*, and Gram-positive bacteria, including *S. aureus*, *S. epidermidis*, and *S. pyogenes*. The solvent-exposed cationic lysines present on the surface of the fibrils engage with negatively charged bacterial cell surfaces, leading to the disruption of membranes. A second generation was constructed through substitution of some lysine residues by arginine residues, MARG1, and PEP6R hydrogels, which presented activity against methicillin-resistant *Staphylococcus aureus* (MRSA) and *P. aeruginosa* [36,37]. Similarly, Liu et al. designed a peptide-based AMH possessing peptide (KIGAKI)_3_-NH_2_ with antibacterial activity against Gram-positive bacteria, which, in response to external stimuli such as pH, ionic strength, or heat, self-assembles into a hydrogel with a cationic surface [38]. Zhou et al. developed an AMH based on the AMP ε-poly-L-lysine modified with methacrylamide (EPL-MA) moieties and cross-linked with polyethylene glycol diacrylate (PEGDA) [39]. It had impressive wide-spectrum antimicrobial activity against bacteria and fungi. This can be affixed onto plastic surfaces, providing a coating option for medical devices. Song et al. successfully developed synthetic polypeptide hydrogels with antibacterial properties by cross-linking poly(Lys)_x_(Ala)_y_ copolymers using six-armed N-hydroxysuccinimide (NHS)-terminated polyethylene glycol (PEG) [40]. It exhibited significant antibacterial activity against *E. coli* and *S. aureus*. Their study also demonstrated that a specific formulation, poly(Lys)_60_(Ala)_40_, exhibited excellent mammalian cell adhesion and proliferation capabilities, along with significant antibacterial activity, highlighting its potential as a wound-healing hydrogel. Bai et al. developed an amphiphilic peptide A_9_K_2_ with the ability to inhibit both Gram-positive and Gram-negative bacterial strains [41]. The sol-gel transition was catalyzed either by lysyl oxidase (LO) in fetal bovine serum (FBS) or plasma amine oxidase (PAO). The enzymatic hydrogel formed by A_9_K_2_ exhibited favorable biocompatibility and demonstrated remarkable selectivity by promoting the adhesion and spreading of mammalian cells. These investigations have presented the potential application of AMPs as antibiotic agents in healthcare settings. However, significant challenges remain, primarily regarding the instability and easy degradation of AMPs. Further research is required to determine whether AMPs can be effectively preserved within hydrogels for extended periods, necessitating additional studies.

#### 2.1.2. Amphoteric Ion Hydrogels

Amphoteric ion hydrogels are synthetic mimics (polymers) of AMPs and operate similarly by employing electrostatic interactions due to the presence of both acidic and basic groups. These interactions enable the hydrogel polymers to bind with the anionic bacterial membrane. The resultant amphiphilic interactions disrupt the membrane structure, ultimately resulting in cell death [42]. Norbornene is a bicyclic compound that can undergo polymerization to form a cross-linked network, resulting in the formation of a poly(norbornene) hydrogel, which is a cationic polymer [43]. Specific modifications in norbornene monomer, such as conjugation with thiol, PEG, or hyaluronic acid, increases antimicrobial activity and biocompatibility [44,45,46]. Similar synthetic cationic polymers have been developed, such as poly(acrylate)s, poly(acrylamide)s, poly-β-lactams, and poly(carbonate)s [47,48,49,50,51,52,53,54].

Poly(vinylpyrrolidone) (PVP) hydrogels showed good cytocompatibility with human oral mucosa stem cells (hOMSCs) in direct contact as well as PVP-coating for cylindrical polyurethane scaffold [55,56,57]. Poly(carboxybetaine) (pCB) and poly(sulfobetaine) (pSB) are zwitterionic polymers that present exceptional swelling capacity in salt solutions, which highlights their potential as dressings for chronic wounds with high exudate levels [58,59,60]. Amphiphilic 9-fluorenylmethoxycarbonyl (Fmoc) amino acids/dipeptides display antibiofilm and anti-inflammatory properties [61,62]. Moreover, after self-assembly, the Fmoc amino acids form potent antibacterial hydrogels with high rigidity and stability against both Gram-positive and Gram-negative bacteria [63,64]. Similarly, Dutta et al. synthesized cholesterol-based amino acid-containing hydrogels, which were also amphiphilic [65]. To incorporate broad-spectrum antibacterial properties, the hydrogels were utilized as a medium for in situ synthesis of silver nanoparticles (AgNPs) using sunlight. The resulting amphiphile-AgNP soft nanocomposite demonstrated remarkable bactericidal activity against both Gram-positive and Gram-negative bacteria.

#### 2.1.3. Polysaccharide-Based Hydrogels

Polysaccharides are the most common natural polymers, and some of them have inherent antimicrobial activity. Polysaccharides, which are complex polymers derived from diverse renewable sources, offer excellent biocompatibility, non-toxicity, biodegradability, abundance in nature, higher water retention, and cost-effectiveness [66,67]. Polysaccharides are isolated from organisms such as plants (cellulose, guar gum, locust bean gum seeds, and starch), algae (alginate, agarose, and carrageenan), animals (chitosan, hyaluronic acid, chondroitin sulfate, collagen, and fibrin), and microbes (dextran, xanthan gum, and gellan gum) [12,68]. However, polysaccharide-based hydrogels are inferior to synthetic hydrogels in terms of mechanical properties and stability [69].

Among all the antimicrobial polysaccharide-based hydrogels, chitosan is the most commonly used due to its unique properties. Chitosan is the deacetylated form of chitin, which is naturally obtained from exoskeletons of crustaceans, insects, and fungi [70]. Chitosan is a linear polysaccharide composed of glucosamine and N-acetylglucosamine, which in part mimics the ECM and cartilage tissue. The deacetylation of chitin increases the amount of amino groups and, therefore, aqueous solubility, bio-compatibility, and biodegradability [71]. It is the only cationic polymer in nature, whereas the previous polysaccharides mentioned are neutral or anionic in charge [8]. The antibacterial features of chitosan may be a result of its association with anions on bacterial cell walls, which inhibits biosynthesis and interferes with transport across cell walls, killing bacteria [72]. Chitosan-based hydrogels can be either chemically or physically cross-linked. The former presents more mechanical stability, whereas the latter can be adjusted more easily to be stimuli-responsive [73].

Hyaluronic acid (HA) is a linear polysaccharide found in ECM of connective or epithelial tissue. There are different options of HA-based hydrogels, and the large majority are designed for wound healing due to the biocompatibility, different cross-linking density to adjust the dosage, and hydrophilicity [74]. For example, Watson et al. designed a gentamicin-loaded HA-based hydrogel for wound dressing, showing antimicrobial activity against Gram-positive and Gram-negative bacteria [75]. However, Zhu et al. synthesized an injectable elastin-like HA-based hydrogel for cartilage regeneration [76]. Cellulose is the most important component of the plant cell wall, and it lacks antibacterial activity [77]. However, bacterial cellulose does exhibit antimicrobial properties, and Zhang et al. obtained it from *Acetobacter xylinumis*, which is active against *E. coli* and *S. aureus* [78,79]. Another example of polysaccharide-based hydrogels with antibacterial properties is gelatin, a derivative of collagen [79,80,81].

It is worth it to mention that most polysaccharide-based hydrogels can be loaded with antimicrobial agents and acquire effective antimicrobial activity, as we review in the next section.

### 2.2. Antimicrobial Agent-Loaded Hydrogels

As we previously reviewed, antimicrobial hydrogels possess good inherent antimicrobial properties and are biocompatible. However, when loaded with antimicrobial agents, their efficacy is increased while allowing the dose of antimicrobial agents to be minimized and resistance to emerge. In addition, antimicrobial agents are released directly at the infected site, avoiding systemic toxicity. There exist five main groups of antimicrobial agents incorporated into hydrogels: antibiotics, biological extracts, metal nanoparticles, and AMPs [82,83,84,85].

#### 2.2.1. Antibiotic-Loaded Hydrogels

Local antibiotic administration has gained attention by delivering an adequate bactericidal dose directly to the infection site without excessive systemic toxicity [86,87]. Hydrogels, as a local administration matrix, offer a high surface area-to-volume ratio and structural controllability, enabling selective drug release at desired sites while maintaining high water content and biocompatibility. Antibiotic-loaded hydrogels offer advantages over conventional antibiotics, including targeted drug delivery, sustained release, reduced resistance development, increased efficacy, improved patient compliance, application in wound care and tissue engineering, and reduced risk of allergic reactions [88,89]. They maintain therapeutic drug levels at the infection site, minimizing systemic side effects. The antibiotics most commonly used are ciprofloxacin, gentamicin, vancomycin, nitroimidazoles, and sulfanilamides [90]. However, they are less studied for clinical use due to the risk of developing bacterial resistance. Ciprofloxacin is the gold standard for topical application in skin or eye infection. It acts through binding and inhibition of DNA gyrase [91]. Sharma et al. developed a chitosan-based hydrogel crosslinked with PEG and loaded with ciprofloxacin and bovine serum albumin (BSA) protein, which mimics a growth factor [92]. The result was an effective, injectable, and self-healing AMH with sustained drug release and potential proteins/growth factors that accelerate the healing process. Ciprofloxacin-encapsulated graphene-silk fibroin macromolecular hydrogels have been designed for burn wound injury and ciprofloxacin and ginsenoside Rh2-loaded poly (lactic-co-glycolic acid)-microsphere thermo-sensitive hydrogel to treat *S. aureus* skin infections [82,93]. Lastly, Giglio et al. designed two electrosynthesized hydrogel coatings loaded with ciprofloxacin that are effective for preventing titanium implant-associated infections frequently related to orthopedic surgery [94].

#### 2.2.2. Biological Extract-Loaded Hydrogels

Biological extract-loaded hydrogels combine the inherent antimicrobial properties of biological extracts or natural compounds from animals and plants with the unique characteristics of hydrogel materials, resulting in versatile platforms for combating microbial infections. The most common biological extracts are herbal extracts, curcumin, essential oils, and honey [95]. These extracts typically contain a wide range of bioactive molecules, such as phytochemicals, peptides, or proteins, that exhibit antimicrobial properties against a broad spectrum of pathogens [96,97]. By loading them into hydrogels, the antimicrobial activity can be preserved and harnessed for sustained release. The porous structure of hydrogels allows for the efficient encapsulation and protection of the loaded extracts or compounds, facilitating their controlled release over time and offering potential biocompatibility and reduced toxicity [98]. The extensively researched groups of chemical compounds originating from biological extracts encompass alkaloids, flavonoids, terpenoids, tannins, and polyphenols [99,100]. 

Some examples of herbal extracts employed in AMH synthesis are the ethanolic and methanolic extracts of Eupatorium glutinosum loaded into a cellulose hydrogel, which displayed, in addition to antimicrobial activity, antioxidative and antihemolytic activity [101]. Lin et al. developed a gelatin and carboxymethyl cellulose-based hydrogel that incorporates several biofunctional extracts, including green tea, Zingiber officinale Rosc, Phyllanthus emblica, and salicylic acid, that present anti-inflammatory, anti-irritant, and antibacterial properties [83]. This gel was suitable for the treatment of acne vulgaris. Gavan et al. designed a carbomer hydrogel loaded with ethanolic extracts of Rosmarinus officinalis aerial parts, Achillea millefolium, and Calendula officinalis flowers [102]. The hydrogel with extract of aerial parts of R. officinalis and the one that incorporates the blend of extracts had good antimicrobial activity and therefore are proposed as novel wound-dressing materials. It is worth it to mention that the ethanolic extracts decrease the consistency, firmness, and adhesiveness of the hydrogel [102]. Synthetic PVA hydrogels have also been tested by loading them with Calendula officinalis extract, which improves the adhesiveness and was effective against both Gram-positive and Gram-negative bacteria [103]. Curcumin, obtained from the roots of Curcuma longa, is a hydrophobic polyphenolic compound with remarkable bioactive properties, and promising results have been obtained by loading it into chitosan or PVA hydrogels [104,105,106,107]. Some essential oils are derived from plants such as lavender, thyme, peppermint, tea tree, rosemary, cinnamon, eucalyptus, and lemongrass [108]. They present antimicrobial properties, and attaching them to hydrogels increase their stability, controlled release, and antimicrobial efficacy [109]. An example is the gelatin hydrogel loaded with microdroplets of rosemary and orange essential oils [110]. Finally, honey exhibits antimicrobial properties attributed to its low pH, low water activity, and the presence of hydrogen peroxide, flavonoids, phenolic compounds, and defensin-1 [84,111]. Some examples are PVP or carboxymethyl cellulose hydrogels loaded with honey, which are effective against S. aureus and E. coli in wound healing [112,113].

#### 2.2.3. Metal Nanoparticle-Loaded Hydrogels

Currently, the most employed metal nanoparticles (NPs) are silver NPs (AgNPs), gold NPs (AuNPs), and zinc oxide NPs (ZnONPs) [21]. The antibacterial effects of silver nanoparticles involve the release of silver ions, which adhere to the cell membrane, disrupt the bacterial envelope, interfere with enzyme activity, generate reactive oxygen species (ROS), and disrupt DNA replication and protein synthesis [114,115]. A wide range of hydrogels incorporating AgNPs exists, including chitosan, chitosan with dextran, chitosan-grafted PVA, and carbopol-based hydrogels [85,116,117,118,119,120]. However, more research is needed to propose the most efficient one [121].

There is much interest in AuNPs due to controlled geometrical, optical, and surface chemical properties and the increasing number of biomedical applications under study [122]. AuNPs exert antimicrobial effects by disrupting bacterial metabolism, inhibiting ribosome function, and interacting with proteins and DNA. They generate oxidative stress, leading to cell damage and death, while demonstrating lower toxicity to mammalian cells [123,124]. However, AuNps are usually loaded, in combination with AgNPs, to hydrogels [125]. An interesting application is the use of these NPs for bone tissue engineering. Ribeiro et al. developed a silk fibroin/nanohydroxyapatite hydrogel loaded with AgNPs and AuNPs, which presented antimicrobial activity against both Gram-positive and Gram-negative bacteria and cytocompatibility with osteoblastic cell lines [126]. Finally, Fmoc-based hydrogels with AuNps or AuNPs/ciprofloxacin are being investigated for the electrochemical detection of the neurotransmitter dopamine in biological fluids [127,128].

Lastly, ZnONPs exhibit antibacterial activity against both Gram-positive and Gram-negative bacteria thermoresistant spores [129]. It seems that the mechanism is similar to that of silver and gold NPs due to an increase in ROS production [130,131]. The most studied is the alginate hydrogel loaded with ZnONPs, which shows excellent antimicrobial activity against *E. coli*, *S. aureus*, *Candida albicans*, and MRSA [132,133]. Bajpai et al. synthesized a ZnONP-loaded hydrogel made of polyacrylate and gum Arabic [134].

#### 2.2.4. AMP-Loaded Hydrogels

AMPs exhibit selectivity for bacteria and safety for mammalian cells [135,136]. Moreover, AMPs do not develop resistance as fast as antibiotics and represent a promising alternative for use alone or in combination with hydrogels [137]. However, in their free state they have a very low half-life, from minutes to a few hours. Therefore, binding AMPs to the hydrogel allows them to retain their antimicrobial activity and targets the infected site directly. Encapsulation in hydrogels allows for controlled and sustained release of AMPs, maintaining steady concentrations at the infection site. Hydrogels shield AMPs from enzymatic degradation, reduce cytotoxicity to healthy cells, and prevent AMP aggregation [138]. The hydrogel matrix provides a favorable microenvironment for AMPs, ensuring proper folding and stability [139]. Enhanced adhesion and retention of hydrogels at the infection site optimize AMP effectiveness. Rezaei et al. attached the AMP Piscidin-1 to a thermo-responsive chitosan hydrogel [140]. It presented antibacterial behavior against resistant *A. baumannii*, excellent biocompatibility, and controlled release of the AMP and water uptake, placing it as a promising candidate for wound dressing. Another example is the AMP RRP9W4N, which was incorporated into an amphiphilic synthetic hydrogel based on Pluronic F127, a copolymer of ethylene and propylene oxide [96]. The result was a mesoporous hydrogel (pore size between 2 nm and 50 nm), consisting of a cross-linked lyotropic liquid crystal. It exhibited antibacterial activity across a wide range, including Gram-positive, Gram-negative, and antibiotic-resistant bacteria, and AMP stability in serum and antibacterial activity was notably increased.

## 3. Translational Applications

### 3.1. Drug Delivery Systems

Hydrogels also act as highly beneficial biocompatible drug delivery systems due to their porosity and compatibility with the aquatic environment. Due to their versatile nature, which allows them to be shaped into diverse physical forms such as films, slabs, microparticles, and nanoparticles, hydrogels find extensive applications in the biomedical field [141]. In swelling-controlled drug release from hydrogels, drugs are dispersed within a glassy polymer that exhibits swelling behavior upon contact with a biofluid. This process is also known as anomalous transport because it combines the processes of diffusion and swelling to enable drug release [142]. The gradient between the dispersed drug in the hydrogel and its surrounding environment allows the active ingredient to diffuse from the region of higher concentration in the hydrogel to a lower one.

The development of hydrogels for hydrophobic drug delivery could provide patients and clinicians with a number of benefits. These hydrogels have mostly been administered in oral, subcutaneous, and transdermal modes of administration [142]. Although there have been some difficulties with employing hydrogels for drug administration, ongoing advancements are being made to find the hydrogel design that is best suited for various drug delivery applications [143,144].

#### 3.1.1. Wound Healing

Hydrogels may have a crucial benefit for wound management, as their antimicrobial action may prevent or delay the development of microbial infections, which are a major obstacle to wound healing and one of the main causes of chronic wounds failing to heal and requiring complex treatment [145,146,147,148,149]. AMHs play a role in activating neutrophils and macrophages to initiate the healing process, inhibiting metalloproteinases and controlling the oxidation–reduction environment [150,151,152].

#### 3.1.2. Tissue Engineering and Regeneration

Hydrogels have more recently been used in tissue engineering, where they can be used as space fillers, as vehicles for the delivery of bioactive compounds, or as three-dimensional structures that arrange cells and provide stimuli to assure the creation of a needed tissue [150]. AMHs offer several advantages in tissue-engineering applications, including their biocompatibility, tunable properties, and ability to provide a suitable microenvironment for cellular activities [14]. In bone tissue engineering, antimicrobial hydrogels are utilized to develop scaffolds for bone regeneration. These hydrogels can be loaded with antimicrobial agents such as silver nanoparticles or antibiotics, which effectively inhibit bacterial growth while promoting the attachment, proliferation, and differentiation of bone-forming cells [153]. Wange et al. introduced hydroxyapatite microspheres to gelatin methacryloyl hydrogel [154]. In contrast, for cartilage regeneration, the AMHs employed are based on hyaluronic acid and elastin [76,155]. Furthermore, antimicrobial hydrogels have been employed in dental tissue engineering, particularly in the development of antibacterial root canal fillings. These hydrogels can release antimicrobial agents within the root canal system to eliminate or suppress bacterial colonization, preventing reinfection and promoting proper healing [156,157]. For example, in root canal disinfection, AMHs have been loaded with diclofenac, chlorhexidine, and metronidazole or silver ions [158,159,160]. In dental pulp regeneration, fibrin or chitosan hydrogels have been loaded with clindamycin-loaded poly (D,L)-lactic acid NPs or polyhexamethyleneguanidine hydrochloride (PHMB), respectively, with promising results in vitro [161,162].

#### 3.1.3. Oral Administration

Antimicrobial hydrogels represent a promising strategy for oral drug delivery to effectively combat diverse oral infections, including periodontitis, dental caries, and oral candidiasis [163,164]. These hydrogels can be formulated as gels or mucoadhesive patches, enabling them to adhere to the oral mucosa, thereby facilitating sustained drug release and localized action. Notably, in the context of periodontal diseases, antimicrobial agents such as antibiotics can be incorporated into the hydrogel matrix [165,166]. Through its adhesion to inflamed gingival tissues, the hydrogel ensures prolonged and direct contact with the infected area, thereby preventing premature drug washout by saliva. This targeted delivery approach enhances therapeutic efficacy while concurrently minimizing systemic side effects [167,168]. The application of antimicrobial hydrogels in oral drug delivery holds significant potential for advancing the treatment of oral infections, potentially improving patient outcomes and overall oral health.

#### 3.1.4. Intranasal Administration

Intranasal drug delivery represents a viable application of antimicrobial hydrogels for the treatment of respiratory infections and sinusitis resulting from bacterial or fungal pathogens [169,170]. By incorporating antimicrobial agents, these hydrogels can be formulated as nasal sprays or gels. Following administration, the hydrogel undergoes gelation within the nasal cavity, leading to the sustained release of antimicrobial drugs at the precise site of infection [171,172]. Leveraging its bioadhesive properties, the hydrogel fosters prolonged drug retention on the nasal mucosa, thereby facilitating enhanced drug penetration and improved therapeutic outcomes [173,174]. This approach holds significant promise in combatting respiratory infections and sinusitis, offering potential benefits in terms of targeted drug delivery, reduced systemic exposure, and the mitigation of adverse effects.

#### 3.1.5. Intravaginal Administration

The intravaginal application of antimicrobial hydrogels offers a promising therapeutic strategy for addressing vaginal infections, including bacterial vaginosis and vulvovaginal candidiasis [175]. These hydrogels can be designed to maintain an appropriate pH environment, ensuring optimal drug activity and enabling the controlled release of antimicrobial agents. Upon administration, the hydrogel adheres to the vaginal mucosa, facilitating the sustained release of antimicrobial agents [176]. This prolonged release allows for effective combating of infecting microorganisms while promoting the healing of vaginal tissues. Furthermore, the mucoadhesive nature of the hydrogel enhances its retention within the vaginal canal, thus reducing the frequency of reapplication and potentially enhancing patient compliance [177].

### 3.2. Contact Lenses

The investigation of hydrogel technology has significantly impacted the daily lives of millions of people through biomedical applications. One of the most notable contributions of hydrogels to modern life, soft contact lenses, led to the development of a new class of optically adjustable soft materials [178]. There are many materials used in contact lenses nowadays. For example, making hydrogels using macromonomers, which are frequently non-toxic, could potentially eliminate the requirement for purification [179]. According to some research, a novel class of optically clear silicone thermoplastic hydrogel materials could be used to make contact lenses [180,181]. The polymers’ general formula includes a section made of silicone and produced from polyciliate that is joined by hydroxyl or amino groups [182]. In addition, among the variety of their applications in vision correction, soft contact lenses may also be utilized to administer medications to the eye [183,184]. The use of these polymers in contact lenses is already well established.

### 3.3. 3D Printing

In the biomedical field, hydrogel films have proven to be effective photonics devices for detection and sensing applications. These films are often used in tabletop laboratory investigations rather than being directly incorporated into biological tissues [185]. Similar to other surface-based assays like physiologically relevant chemicals, lateral flow chips, and fluids, analyte identification can be evaluated in these films [186,187]. Different types of structures have been created for this proposal, adding 3D complexity to the constructs’ optical properties and biological compatibility. Another fabrication method that has become increasingly popular in the field of bio-fabrication is 3D printing [188]. This method makes it possible to incorporate complex shapes and control the precise deposition of various materials and cells, which facilities the replication of the complexity of biological tissue [189,190]. It is not impossible to combine several fabrication techniques, such as optical fiber integration and 3D printing, into structures. Advanced in vitro tissue emulation and real-time monitoring and reporting of pertinent responses through light-based readouts could be combined using this strategy [191].

### 3.4. Biosensing

The analysis of biological markers utilizing a transducing mechanism is called biosensing [192]. This is frequently used for the detection of a wide range of biological targets, including cells, bacteria, viruses, and tiny molecules, including uric acid, glucose, and H_2_O_2_, as well as biomacromolecules, nucleic acids, enzymes, proteins, and peptides. A family of materials known as “smart materials” is capable of reacting to a variety of environmental factors, including temperature, pH, moisture, light, chemical compounds, magnetic or electric fields, and bio-stimuli [193]. In this sense, hydrogels are considered “smart” materials.

Biosensors made of hydrogels are typically used in water environments. Additionally, there is a conflict between the swelling of the hydrogel caused by the fluid and the analysis, which could influence how the analyte levels are assessed [194]. They are used in sensing glucose, nucleic acids, proteins, and enzymes [195,196,197].

### 3.5. Water Purification

The deteriorating environment receives a critical requirement from green chemistry for a sustainable addition to human society [198]. Water pollution in the human environment is the biggest global concern, having a negative impact on many living things and leading to major health problems [199].

**Figure 2:**
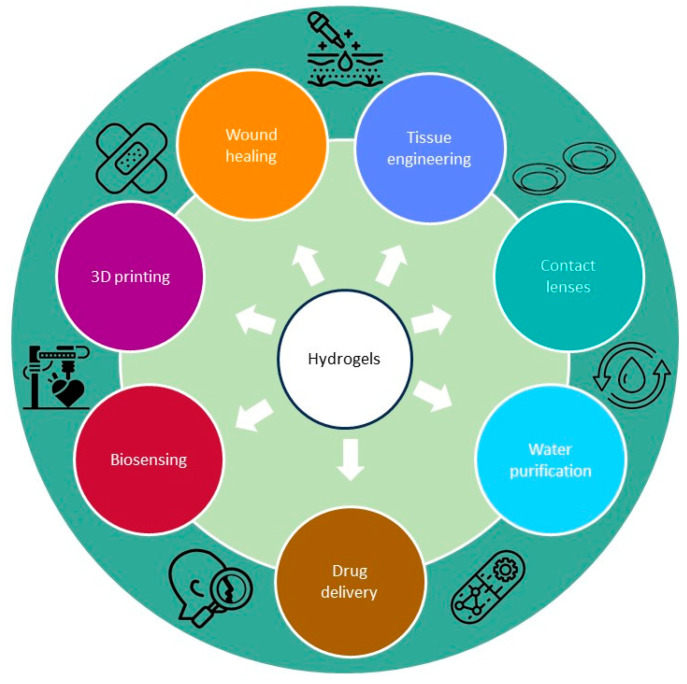
Translational application of antimicrobial hydrogels in biomedicine.

Because of their unique characteristics, hydrogels can be used to purify water. They are three-dimensional, branched polymers with exceptional water absorption capabilities [200]. The water absorption characteristic of hydrogels is important, as it allows them to absorb and retain large amounts of water, increasing the contact time between water and antimicrobial agents, thereby enhancing their efficiency in purifying water and ensuring safe drinking water supplies [201,202]. The hydrogel’s stretchability, pliability, and porousness properties are sensitized to its capacity to absorb water [203]. Figure 2 and Table 1 provides an overview of antibacterial hydrogels’ most promising potential uses.

## 4. Conclusions and Future Perspectives

In conclusion, the reviewed literature highlights the rapid evolution and promising potential of antimicrobial hydrogels in the medical management of infectious diseases. Their unique physicochemical and biological properties, coupled with the ability for targeted drug delivery, make them an attractive solution for combating infections and addressing the challenges associated with antibiotic resistance. However, further research is necessary to optimize the design, formulation, and efficacy of antimicrobial hydrogels, enabling their widespread clinical implementation and ultimately improving patient outcomes. Furthermore, exploring multi-modal approaches that combine antimicrobial hydrogels with complementary therapeutic approaches may enhance their effectiveness in combating infectious diseases. Rigorous preclinical and clinical investigations are essential to establish the safety and efficacy of antimicrobial hydrogels before their routine integration into medical practice. As the field continues to advance, antimicrobial hydrogels hold significant promise as an innovative and impactful approach to mitigate the global threat of antimicrobial resistance.

## Figures and Tables

**Table 1:** Translational applications of antimicrobial hydrogels (AMHs).

Translational Applications		Purpose	Examples of AMHs	References
Drug delivery systems	Wound healing	Provide a controlled and sustained release of antimicrobial agents to combat infections while simultaneously promoting tissue repair and regeneration	Hyaluronic acid-based Zn^2+^-loaded cellulose-based AgNPs-loaded chitosan grafted polyvinyl alcohol-based	[74,116,151]
	Tissue engineering	Create an environment that supports cell growth and tissue regeneration while preventing infections with the controlled release of antimicrobial agents	Ag-doped hydroxyapatite NPs-loaded gelatin methacryloyl-based Hyaluronic acid/elastin-like polypeptide hybrid	[154,155]
	Oral administration	Deliver targeted and sustained release of antimicrobial agents to treat oral infections	Poly(vinyl alcohol)-based Methacrylated gelatin andpoly(ethylene glycol) diacrylate-based	[165]
	Intranasal administration	Provide localized and sustained delivery of antimicrobial agents for effective treatment of respiratory infections	Carbopol and hydroxypropyl-β-cyclodextrin-based Amoxicillin trihydrate-loaded bovine serum albumin NPs-loaded poloxamer-based	[169,173]
	Intravaginal administration	Offer targeted and controlled release of antimicrobial agents to effectively treat vaginal infections while promoting tissue healing and minimizing the need for frequent reapplication	Clotrimazole-loaded poloxamer-based	[177]
Contact lenses		Provide a protective and hygienic surface, reducing the risk of microbial infections and promoting ocular health	Silicone-based Epigallocatechin gallateloaded starch-based Mel4 peptide-coated silicone-based	[180,183,184]
3D printing		Develop biocompatible and infection-resistant constructs for biomedical applications	Alginate-based	[190]
Biosensing		Enhance the sensitivity and specificity of biosensors	Protease-responsive hydrogels of poly(ethyleneglycol) diacrylate Estradiol-sensitive carboxylated p(NIPAM) Glucose-sensitive of chitosan and dextran	[196,197]
Water purification		Remove and inhibit the growth of microorganisms, enhancing the safety and quality of drinking water	Polyacrylamide/bentonite/graphitic carbon 976 nitride Lignin-containing cellulose nanofibril-reinforced polyvinyl alcohol	[198,200]

## Data Availability

Not applicable.
